# Characterization of T Follicular Helper Cells and T Follicular Regulatory Cells in HIV-Infected and Sero-Negative Individuals

**DOI:** 10.3390/cells12020296

**Published:** 2023-01-12

**Authors:** Bradley Salvatore, Rachel S. Resop, Brent R. Gordon, Marta Epeldegui, Otoniel Martinez-Maza, Begoña Comin-Anduix, Alex Lam, Ting-Ting Wu, Christel H. Uittenbogaart

**Affiliations:** 1 Department of Microbiology, Immunology and Molecular Genetics, University of California Los Angeles (UCLA), Los Angeles, CA 90095, USA; 2 UCLA AIDS Institute, David Geffen School of Medicine, University of California Los Angeles (UCLA), Los Angeles, CA 90095, USA; 3 Department of Obstetrics and Gynecology, University of California Los Angeles (UCLA), Los Angeles, CA 90095, USA; 4 Jonsson Comprehensive Cancer Center (JCCC), University of California Los Angeles (UCLA), Los Angeles, CA 90095, USA; 5 Department of Surgical-Oncology, University of California Los Angeles (UCLA), Los Angeles, CA 90095, USA; 6 Department of Molecular Pharmacology, University of California Los Angeles (UCLA), Los Angeles, CA 90095, USA; 7 Department of Pediatrics, University of California Los Angeles (UCLA), Los Angeles, CA 90095, USA

**Keywords:** T follicular helper cells, T follicular regulatory cells, HIV, flow cytometry

## Abstract

Humoral immune response is important in fighting pathogens by the production of specific antibodies by B cells. In germinal centers, T follicular helper (TFH) cells provide important help to B-cell antibody production but also contribute to HIV persistence. T follicular regulatory (TFR) cells, which inhibit the function of TFH cells, express similar surface markers. Since FOXP3 is the only marker that distinguishes TFR from TFH cells it is unknown whether the increase in TFH cells observed in HIV infection and HIV persistence may be partly due to an increase in TFR cells. Using multicolor flow cytometry to detect TFH and TFR cells in cryopreserved peripheral blood mononuclear cells from HIV-infected and non-infected participants in the UCLA Multicenter AIDS Cohort Study (MACS), we identified CD3^+^CXCR5^+^CD4^+^CD8^−^BCL6^+^ peripheral blood TFH (pTFH) cells and CD3^+^CXCR5^+^CD4^+^CD8^−^FOXP3^+^ peripheral blood TFR (pTFR) cells. Unlike TFR cells in germinal centers, pTFR cells do not express B cell lymphoma 6 (BCL6), a TFH cell master transcriptional regulator. Our major findings are that the frequency of pTFH cells, but not pTFR cells was higher in HIV-infected participants of the MACS and that pTFH cells expressed less CCR5 in HIV-infected MACS participants. Constitutive expression of CCR5 in TFR cells supports their potential to contribute to HIV persistence.

## 1. Introduction

Human immunodeficiency virus type 1 (HIV-1) is the etiologic agent of acquired immunodeficiency syndrome (AIDS), which was first described in the United States at UCLA in 1981 (Gottlieb, M.S.-NEJM, 1981) and soon thereafter by Luc Montagnier and Robert C. Gallo [1,2]. With current combination antiretroviral therapies (cART), HIV-1 infection can be controlled effectively. However, CD4^+^ T cells still harbor HIV-1 integrated genetic material, allowing the virus to persist indefinitely in stable latent reservoirs [3]. Thus, HIV-infected cells are difficult to eradicate completely. Any cell type that allows HIV to persist despite optimal cART and can support replication in the absence or sup-optimal ART is deemed to be an HIV reservoir [4]. This includes resting memory CD4^+^ T cells with integrated virus genome in individuals in the later stage of HIV-1 infection [5]. There are several potential HIV reservoirs that can contribute to HIV persistence: CD4^+^ T cells in the lymph nodes, gut-associated lymphoid tissue, the central nervous system, hematopoietic stem cells, macrophages, and regulatory T cells [4,6].

People living with HIV (PLWH) have low circulating CD4^+^ T cell counts. CD4^+^ T-cell subsets, displaying the co-receptors CXCR4 and CCR5, are susceptible to HIV infection and can contribute to HIV persistence. Previously, T follicular helper (TFH) cells were shown to be infected by SIV and HIV [7]. TFH cells, differently from Th1, Th2, or Th17, are a distinct subset of CD4+ T cells, which were described in the early 2000s [8]. These cells localize to the germinal center (GC) of the B cell follicles and provide T-cell help to B cells using the chemokine receptor CXCR5. In mice, GCs TFH cell differentiation and development are dependent on the master transcriptional regulator, B cell lymphoma 6 (BCL6) [9,10,11]. Mouse and human TFH cells express the chemokine receptor CXCR5, which allows their migration to the B-cell follicles [8]. Additionally, they express inducible T-cell costimulator (ICOS) and programmed death-1 (PD1) [12]. In secondary lymphoid tissues, CXCR5 can be used in combination with BCL6 or PD1 to identify GC TFH cells in mice and humans [12]. HIV-1 infection of GC TFH cells is perilous because B cells will not function properly without T cell help, thus weakening the immune system. Lastly, TFH cells could act as an HIV reservoir and thus allow HIV to persist in the body, leading to HIV resurgence upon discontinuation of cART [4].

In addition to TFH cells, another CD4^+^ CD3^+^ T-cell subset, which develops from thymus-derived fork head box P3 (FoxP3)^+^ precursors, was described in mice and named T follicular regulatory T (TFR) cells [13,14,15]. FOXP3^+^ T regulatory cells maintain immune homeostasis and prevent autoimmunity by inhibiting proliferation and cytokine production of effector T cells [16,17]. In mice, TFR cells have been shown to suppress GC reactions and also to play a role in preventing autoimmunity and exaggerated immune responses [13]. TFR cells suppress the immune system either by interfering with T-cell help to B cells or directly suppressing B-cell function. However, the maintenance of TFH and TFR cells is independent of the GC in humans [14]. TFH and TFR cells are both found in human tonsils [18]. Treatment with CD20 monoclonal antibody rituximab (RTX), dampening the immune system of renal transplant patients, causes significant depletion of B cells in the GCs in the lymph node [18]. However, TFH and TFR cells are still present in the absence of B cells, suggesting that the maintenance of TFH and TFR cells is independent of B-cell activity in GCs [18]. If TFR cells can harbor viral DNA and allow the persistence of HIV, it would be detrimental to the regulation of TFH cells within GCs and may even lead to autoimmune diseases.

Both GC TFH and GC TFR cells can be identified in mouse secondary lymphoid tissues by surface expression of CD3, CD4, CXCR5, PD-1, and ICOS and the transcription factor BCL6 [12,19]. GC TFR cells can be differentiated by the expression of an additional transcription factor FOXP3 [13]. However, unlike in the germinal center, TFR cells in human peripheral blood do not express BCL6 [20]. Due to their similar phenotypic characteristics, it is possible that in the absence of FOXP3 determination the previously identified TFH cell population, acting as an HIV reservoir, also includes TFR cells. Thus, it is still unknown whether TFR cells can contribute to HIV persistence.

We examined TFR cells in peripheral blood mononuclear cells (PBMCs) from HIV-infected and non-infected participants from the UCLA Multicenter AIDS Cohort Study (MACS) using multi-color flow cytometry. PBMCs were stained to immunophenotype peripheral blood TFH and TFR (pTFH and pTFR) cells. First, we compared the phenotypic profile of pTFH and pTFR cells in fresh and frozen healthy PBMCs from anonymous donors in order to assess the effect of freezing on the immunophenotype. Second, we examined TFH and TFR cells in PBMCs from HIV-negative and HIV-infected participants of the MACS.

Although an increase in GC TFH cells in HIV-infected lymphoid tissue has been shown [21], our study was the first report of an increase of pTFH cells in peripheral blood of HIV-infected participants in the MACS. In addition, we found that pTFR cells also expressed a higher level of CCR5 in PLWH than from seronegative participants, making them more susceptible to CCR5-tropic HIV infection and to becoming an HIV reservoir. HIV persistence despite combination antiretroviral therapy (cART) is a challenge in HIV eradication. The ability to identify T-cell subsets, which serve as an HIV reservoir, is an important milestone in developing a cure for PLWH. By eliminating HIV reservoirs, the virus will not persist after cART which may contribute to a functional cure for HIV infection.

## 2. Materials and Methods

### 2.1. Ethics Statement

The use of anonymous peripheral blood mononuclear cells (PBMCs) from healthy donors and de-identified participants of the UCLA Multicenter AIDS Cohort Study (MACS) was reviewed by the UCLA Institutional Review Board (IRB), which concluded that these activities did not involve human subjects, and therefore did not require IRB review or certification.

### 2.2. Experimental Design

Our experimental aim was to immunophenotype pTFR cells expressing the FOXP3 intracellular marker and to differentiate this newly found population from the well-known pTFH cells. We conducted cytometric analyses of PBMCs by multi-color flow cytometry using the following combination of extracellular markers: CD3, CD4, CD8, CXCR5, CD25, CCR5, PD-1, ICOS, and T-cell immunoreceptor with Ig and ITIM domains (TIGIT). We also stained for FOXP3 and BCL6 as the differentiation intracellular markers for TFR cells. We defined pTFH cells as BCL6^+^CD3^+^CXCR5^+^CD8^−^, and pTFR cells as FOXP3^+^CD3^+^CXCR5^+^CD8^−^. We first assessed the integrity of surface marker after freezing/thawing by comparing fresh and short-term (7 days) frozen peripheral blood samples from the same donor. This short-term freezing and then thawing mimicked the conditions of the MACS samples. Then, we utilized our established flow-cytometry panel to investigate pTFH and pTFR cells from MACS samples.

### 2.3. Peripheral Blood Mononuclear Cells

Peripheral blood mononuclear cells (PBMCs) were obtained from anonymous healthy donors from the UCLA Center for AIDS Research (CFAR), Virology Core. Since secondary lymphoid tissues are hard to obtain from PLWH, we chose to use viably cryopreserved PBMCs from the UCLA Multicenter AIDS Cohort Study (MACS). The MACS HIV-infected samples were obtained at various stages of HIV infection, but specimens from AIDS-diagnosed participants were not used in order to not confound the effect of cancer and other opportunistic infections. The MACS peripheral-blood samples are provided to researchers only with a de-identified code. The MACS peripheral blood samples used in this study were obtained from HIV-infected and non-infected participants who were treated with cART and matched for age and visit number, and their lowest CD4 count.

### 2.4. Flow Cytometry

Violet ^TM^510 from Tonbo-Biosciences was used as a viability dye. Cell-surface staining was done as previously described. The intracellular staining procedure was modified from our previously published paper [22]. Intracellular protein staining was combined with surface protein staining as a way to identify pTFH and pTFR cells. First, 4–5 × 10^6^ cells were washed with a staining buffer and then incubated with human Ab serum along with all surface staining antibodies. Surface staining antibodies included: CD3, CD4, CD8, CXCR5, CD25, CCR5, PD-1, ICOS, and TIGIT. Details of antibody fluorochromes and sources can be found in Table 1. Extra antibodies were washed off with the appropriate staining buffer [22]. The cells were then fixed and permeabilized with FOXP3/Transcription Factor Fixation/Permeabilization Concentrate and Diluent (eBioscience, Santa Clara, CA, Cat. No. 00-5521-00) for 30 min at room temperature. Cells were washed twice with Permeabilization Buffer (eBioscience, Cat. No. 00-8333-56). Next, cells were stained intracellularly with FOXP3, BCL6, and appropriate IgG control (antibody sources in Table 1). Extra antibodies were washed off with the nanocrystal buffer. Finally, cells were re-suspended in a staining buffer before acquisition on a SORP HTLSRII Analytic Flow Cytometer (BD Immunocytometry Systems). Compensation tubes were not permeabilized, but were fixed with 1% paraformaldehyde.

In all experiments gating was done on live cells followed by lymphocytes and single cells. TFH and TFR cells were distinguished by expression of BCL6 and FOX-P3 (Supplementary Materials Figure S1).

### 2.5. Mass Cytometry-CyTOF

PBMCs from HIV-infected and non-infected MACS participants were immunophenotyped using a Helios Mass Cytometer (CyTOF) [23]. Antibodies conjugated to metal isotopes, including the cell surface markers CXCR5, CD3, CD4, CD8, CD25, CD31, CD45RA, CD69, CCR7, and CD127, were used to distinguish pTFR cells from other T-cell subsets; HIV co-receptors CCR5 and CXCR4; and inhibitory receptors PD1 and TIGIT. Furthermore, intracellular markers, BCL6, FOXP3, BLIMP1, and KLF2, known to be expressed in the follicular T-cell subsets and Ki67, a marker of cellular proliferation, were included in the CyTOF panel. Although PD1 expression was decreased on frozen cells, its level was sufficient to be included in the panel (Table 2). Dead PBMC dead cells were excluded using Cell-ID Cisplatin supplied by Fluidigm.

Samples were analyzed using the OMIQ platform. Events were gated, eliminating time fluctuation, TYPE beads, and doublets. Then, double DNA (Ir^+^) to gate for cells, from those live cells (Cis-Platin negative), and finally T-cell (CD3^+^CD19^−^) events. Inside OMIQ, unsupervised analysis using uniform manifold approximation and projection (UMAPS) and, posteriorly, FlowSom for clustering were used. Supplementary Materials Figure S2A shows the gating strategy before projection/clustering.

### 2.6. Statistical Analysis

All statistical analyses were conducted with GraphPad Prism 5.02 (Company: GraphPad Software, San Diego, CA. All variables are shown as means with standard error of the mean (SEM). Unpaired T-test and Mann–Whitney test were used when appropriate to compare the mean between different populations. A significant *p*-value is equal to or less than 0.05 (*: *p* ≤ 0.05, **: *p* ≤ 0.01, ***: *p* ≤ 0.001, ****: *p* ≤ 0.0001).

## 3. Results

### 3.1. TFR (CXCR5^+^CD3^+^CD4^+^CD8^−^FOXP3^+^) and TFH (CXCR5^+^CD3^+^CD4^+^CD8^−^FOXP3^−^) Cells Can Be Detected in Fresh and Frozen Peripheral-Blood Lymphocytes from Healthy Individuals with Minimal Phenotypic Changes

**Figure 1:**
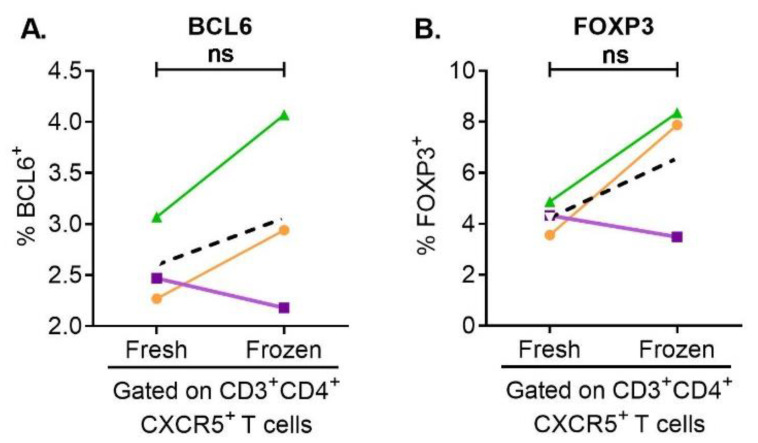
pTFH and pTFR cells were present in fresh and frozen PBMCs from 3 different donors. Using multicolor flow cytometry, we could detect pTFH (**A**) and pTFR (**B**) cells. The expression of BCL6 and FOXP3 did not change significantly upon freezing using unpaired T-test and Mann–Whitney tests (ns: *p* > 0.05).

To evaluate the effect of freezing/thawing on the frequency and immunophenotype of pTFR and pTFH cells within fresh and short-term frozen (7 days) PBMCs from the same healthy donor were tested fresh and after at least one week of cryopreservation. We used multicolor flow cytometry to acquire the data and FCS express to analyze the results. PBMCs were gated on viable CXCR5^+^CD3^+^CD4^+^ cells and the percentage of pTFH and pTFR cells before and after freezing was determined by expression of intracellular BCL6 (Figure 1A), and expression of FOXP3 in pTFR cells (Figure 1B). A lack of statistical significance in the percentages of pTFH and pTFR cells in fresh and frozen PBMCs from the same donor suggests that the numbers of TFR cells was not affected by freezing/thawing, at least after short term storage. Appropriate IgG controls were used for all the experiments.

**Figure 2:**
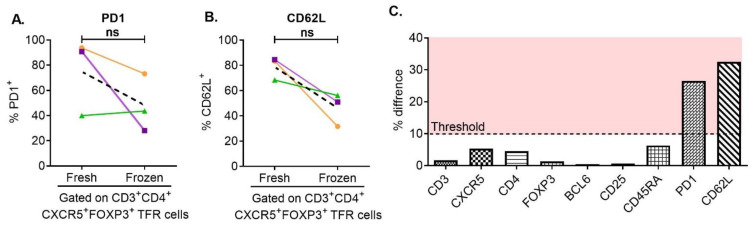
PD-1 and CD62L decreased after short-term freezing. Using multicolor flow cytometry, fresh and frozen PBMCs from 3 different donors were immunophenotyped for pTFH- and pTFR-cell expression of cell-surface markers to assess the effect of freezing/thawing (**A**,**B**). Expression of PD-1 and CD62L was affected by freezing (**C**). The average difference in the expression of each surface marker between fresh and frozen samples is shown. Only PD-1 and CD62L expression changed more than an arbitrarily selected threshold of 10% difference (**C**). (ns: *p* > 0.05).

To examine whether freezing influenced the expression of cell-surface and intracellular markers in PBMCs we evaluated percentages of CD3, CD4, CXCR5, CD25, PD1 and CD45RA and the intracellular markers FOXP3 and BCL6 in fresh and frozen PBMCs from the same donor. As shown in Figure 2 there were no clear differences in expression of the cell surface markers CD3, CD4, CXCR5, CD25, or CD45RA or in the intracellular markers FOXP3 and BCL6 between fresh and frozen PBMCs. However the expression of the cell surface markers PD1 and CD62L was affected by cryopreservation (Figure 2). These data are important as only frozen PBMCs were available for our studies of PBMCs from HIV-infected and non-infected MACS participants.

### 3.2. CD4^+^ T Cells Expressing CXCR5 Can Still Be Detected in Long-Term-Frozen Human PBMCs

**Figure 3:**
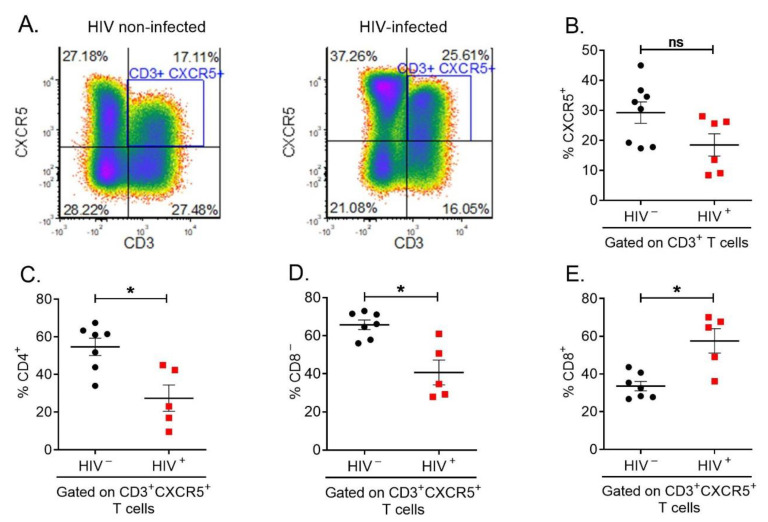
CD4^+^CXCR5^+^ T cells can be detected in long-term-frozen human PBMCs. Previously frozen and then thawed PBMC samples from the MACS were stained with antibodies against CD3, CXCR5, CD4, and CD8. (**A**) Representative flow-plots from an HIV-non-infected individual (left) and an HIV-infected individual (right), showing CXCR5^+^ CD3^+^ population within total human PBMCs. The cursors were set in each sample based on the IgG control (**B**) Quantification of CXCR5 expression within the CD3^+^ T cells. HIV− (*n* = 8, mean ± SEM), HIV+ (*n* = 6, in red, mean ± SEM), Mann–Whitney U test, (*p* = 0.0593, ns). (**C**) Quantification of CD4 expression within the CXCR5^+^ CD3^+^ T cells. (**D**) Quantification of CD8^−^ population within the CXCR5^+^CD3^+^ T cells. (**E**) Quantification of CD8 expression within the CXCR5^+^ CD3^+^ T cells. (**C**–**E**) HIV− (*n* = 8, mean ± SEM), HIV+ (*n* = 5, in red, mean ± SEM), Mann–Whitney U test (* *p* < 0.05).

We next performed a phenotypic comparison of the pTFR cell population between HIV-infected and non-infected participants from the MACS. Because CXCR5 expression was slightly affected by freezing and thawing (Figure 2C), we first assessed expression of CXCR5 in the MACS samples. Figure 3A showed a distinct population of CXCR5^+^CD3^+^ T cells within the live lymphocytes singlet in HIV-non-infected (left) and HIV-infected (right) participants. There was a mean of 28.63% CXCR5^+^CD3^+^ T cells in HIV-non-infected participants, and there was a mean of 19.28% CXCR5^+^CD3^+^ T cells in HIV-infected participants (Figure 3B). These data showed a decreasing trend of CXCR5^+^CD3^+^ T cells in HIV-infected participants, but this was not statistically significant (*p* > 0.05).

Additionally, previous studies have shown that CD4 receptor is down-regulated by HIV-1 infection [24]. In order to observe the effect of HIV infection on the CD4 receptor, we compared the proportion of CD4^+^ T cells and CD8^–^ T cells gated on CD3^+^CXCR5^+^ (Figure 3C,D). The mean proportion of CD4^+^ T cells decreased from 53.73% in HIV-non-infected to 28.71% in HIV-infected participants (* *p* < 0.05) (Figure 3C). The mean proportion of CD8^–^ T cells decreased from 64.63% in HIV-non-infected to 42.14% in HIV-infected participants (* *p* < 0.05) (Figure 3D). The similar decrease in HIV-infected participants for both CD4^+^ T cells and CD8^–^ T cells showed that CD8^−^ T cells can be used to approximate CD4^+^ T cells to select T cells in which CD4 receptor is down-regulated. Interestingly, the proportion of cytotoxic CXCR5^+^ CD8^+^ T cells increased from 34.74% in HIV-non-infected to 55.86% in HIV-infected participants (* *p* < 0.05) (Figure 3E). Overall, we demonstrate that CXCR5^+^CD3^−^ T cells can be detected in long-term-frozen PBMCs, and CD8^–^ gating strategy is an effective way to include T helper cells with down-regulated CD4 receptor due to HIV infection.

We also searched for pTFR cells using higher dimensional analysis by mass cytometry. Frozen PBMCs from non-infected and HIV-infected participants on cART were stained with a combination of >20 metal-conjugated antibodies to examine for the presence of pTFR cells. As shown in Supplementary Materials Figure S2B, pTFR cells were detected in PBMCs from both subjects, which provides us with a full spectrum of immunophenotype. pTFH cells were also present but are not displayed

### 3.3. In Peripheral Blood, There Is a Trend of an Increased Frequency of pTFH Cells in HIV Infection, Which Is Not Observed in pTFR

**Figure 4:**
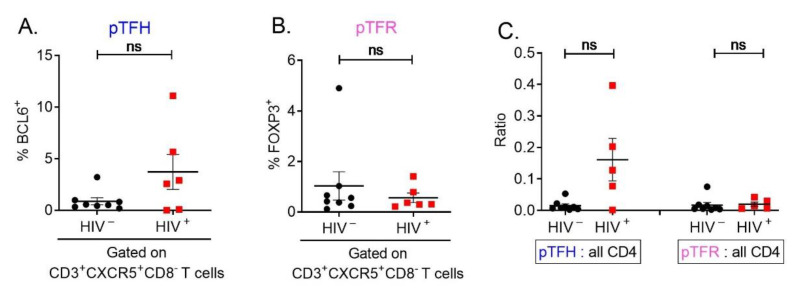
pTFH-cell frequency increases upon HIV infection, but pTFR-cell frequency does not. Viably frozen PBMCs from the MACS were thawed and immunophenotyped for pTFH and pTFR cells. (**A**,**B**) pTFH cells were characterized as FOXP3^-^BCL6^+^CD3^+^CXCR5^+^CD8^−^CD4^+^ and pTFR cells as FOXP3^+^CD3^+^CXCR5^+^CD8^−^CD4^+^. (**C**) pTFH and pTFR cells frequency was compared relative to total CD4^+^ T cells to control for individual variation in the number of T cells. (ns: *p* > 0.05).

To identify pTFH and pTFR cells in PBMC samples we selected CD3^+^CXCR5^+^CD8^−^ T cells from our flow cytometry data. Then, BCL6 and FOXP3 were used to select for pTFH and pTFR cells, respectively. In human PBMCs, pTFH cells uniquely express BCL6, and pTFR cells uniquely express FOXP3 (Figure 4A,B). These results show that pTFR cells and GC TFR cells are phenotypically different in BCL6 expression.

It is unknown whether the reported increase in pTFH cells during HIV infection is due to a decrease in pTFR cells. Thus, we investigated whether HIV infection had an effect on the frequency of pTFH and pTFR cells. While there was a trend of a greater proportion of BCL6^+^CD3^+^CXCR5^+^CD8^−^ pTFH cells in HIV-infected participants than in non-infected participants (Figure 4C), the proportion of FOXP3^+^CD3^+^CXCR5^+^CD8^–^ pTFR cells was similar (Figure 4B). In order to minimize the effect of individual variation in the number of CD4^+^ T cells, the frequency of pTFH or pTFR cells was assessed by the ratio of pTFH and pTFR cells divided by total number of CD4^+^ T cells. Relative to all CD4^+^ T cells, the frequency of pTFH cells showed an apparent trend to be higher in HIV-infected than in non-infected participants (*p* > 0.05). (Figure 4A). An increase in GC TFH cells has been reported in lymph nodes [25] and spleens [26] of chronically infected PLWH and may contribute to the dysregulation of B-cell function such as hyper-gammaglobulinemia, polyclonal activation, and a decrease in antigen-specific antibody production. In addition, the frequency of pTFR cells relative to all CD4^+^ T cells remained similar in HIV-infected and non-infected participants (Figure 4C), which is in contrast to the increase in TFR cells observed in spleen and lymph nodes of PLWH [26,27]. Based on these results, an increase in pTFH cells in peripheral blood of HIV-infected MACS participants was not due to the increase in pTFR cells.

### 3.4. The HIV Co-Receptor CCR5 Expression on pTFRCells in HIV-Infected MACS Participants Is Increased

**Figure 5:**
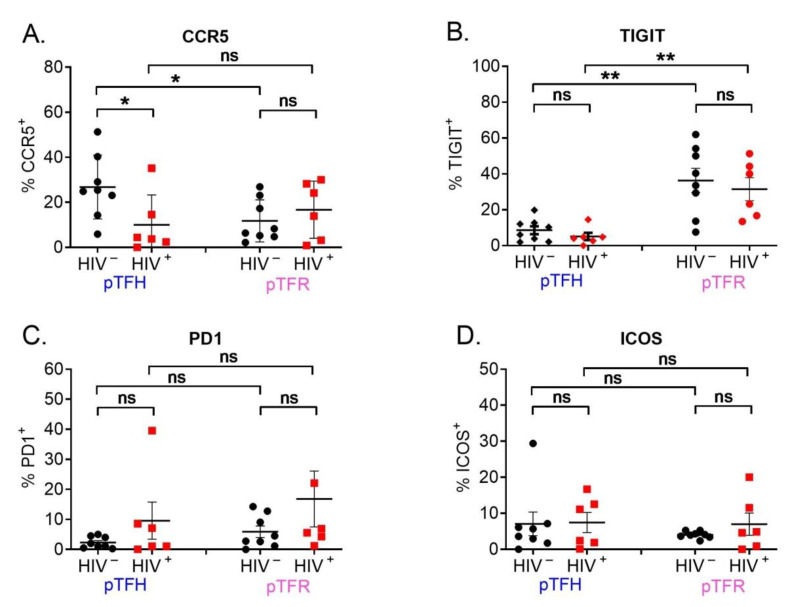
Expression of surface markers of pTFH and pTFR cells. (**A–D**) Quantification of the percentage of cells expressing CCR5, TIGIT, PD1, or ICOS on pTFR and pTFH cells, respectively. HIV− (*n* = 8, mean ± SEM). HIV+ (in red, *n* = 6, mean ± SEM) (Mann–Whitney U test). (**A**,**B**) In both pTFH and pTFR cells, the percentage of TIGIT-expressing cells was similar in HIV-infected and non-infected individuals. In HIV-infected and non-infected individuals, the expression of TIGIT was higher in pTFR than in pTFH cells. (**C,D**). In both pTFH and pTFR cells, the percentage of cells expressing PD1/ICOS was similar in HIV-infected and non-infected participants of the MACS. (ns: *p* > 0.05, *: *p* ≤ 0.05, **: *p* ≤ 0.01).

We aimed to identify the contribution of pTFR cells to HIV persistence by comparing the phenotypic characteristics of pTFR cells between HIV-infected and non-infected participants. GC TFH cells have been shown to be highly susceptible to HIV infection and can contribute to HIV persistence [7,12]. Both pTFH and pTFR cells expressed CCR5, indicating that they are susceptible to CCR5-tropic HIV infection. In pTFR cells, the proportion of CCR5 expression was higher, although not significantly, in HIV-infected participants (*p* > 0.05) while in pTFH cells, the decrease in CCR5 expression was not statistically significant in HIV-infected participants (*p* > 0.05) (Figure 5A).

### 3.5. Changes in Surface Markers on pTFH and pTFR Cells

The presence of bona fide TFH and TFR cells in human blood is controversial, partly due to varied phenotypic definition across studies [28]. In our study, pTFH cells were defined as CXCR5^+^CD3^+^CD4^+^CD8^−^BCL6^+^ cells and pTFR cells were defined as CXCR5^+^CD3^+^CD4^+^CD8^−^FOXP3^+^ cells. In order to effectively discriminate pTFH and pTFR cells by surface markers for functional studies in the future, we aimed to further characterize pTFR and pTFR cells with PD-1, TIGIT, ICOS, CD25, and CD45RA. We also aimed to determine the phenotypic differences between pTFH and pTFR cells in HIV-infected and non-infected MACS participants.

Both pTFH and pTFR cells expressed PD-1, TIGIT, ICOS, CD25, and CD45RA. The percentage of TIGIT-positive cells was significantly higher on pTFR cells than on pTFH cells (** *p* < 0.001) (Figure 5B), agreeing with data in mice indicating that TFR-cell expression of TIGIT was two-fold higher than TFH cells [29]. We found that the percentage of TIGIT on pTFR cells was similar in HIV-infected and non-infected participants (Figure 5B). The expression of PD-1 and ICOS in pTFH and pTFR cells showed a distinct trend of increasing in HIV-infected participants, but this was not statistically significant (Figure 5C,D). Within HIV-infected participants, a wide range of PD-1 and ICOS expression might be due to the different stage of HIV progression. However, previous studies characterized TFH and TFR cells in the lymph nodes using just the surface markers CXCR5 and PD-1 or CXCR5 and CD25. In frozen peripheral blood, we cannot use PD-1 or any other surface marker to reliably distinguish pTFH and pTFR cells without staining for their intracellular markers.

## 4. Discussion

TFR cells were first identified in the GCs of the mouse lymphoid tissue [13] and then in human tonsils [18]. There is little known of pTFR cells and their function in humans. In this study, we aimed to characterize pTFR cells in PBMCs of HIV-infected and non-infected participants from the frozen peripheral-blood lymphocytes from the MACS. Upon freezing, we found that the surface markers PD-1 and CD62L were destabilized in cells from the same PBMC samples because of the stress occurring during the freezing/thawing procedure. CD62L, a lymph-node homing receptor, was not a crucial marker in the identification of pTFR cells, and thus was not included in the immunophenotyping of the MACS samples. However, we kept PD-1 on the staining panel because this marker has been commonly used in combination with CXCR5 and ICOS to identify TFH and TFR cells [30]. Other markers such as CD3, CD4, FOXP3, BCL6, and especially CXCR5 remained unaffected by freezing/thawing.

Similar to TFH cells, the precise identification of TFR cells has been controversial and difficult because of their varied surface phenotypic definition in different reports [30]. It was expected that pTFR cells would be different from their counterpart in the secondary lymphoid tissues. In this study, we showed that CD3^+^CD4^+^CXCR5^+^ follicular T cells in peripheral blood express FOXP3, along with other surface markers such as PD1, CD45RA, ICOS, TIGIT, and CD25. Human pTFR cells, unlike GC TFR cells, do not express BCL6 in peripheral blood [20], indicating a potential difference in function between pTFR and GC TFR cells; or pTFR cells may even be a completely novel population. The lack of BCL6 in pTFR cells reinforced the finding of the origin of GC TFR cells which is reported to be thymic-derived FOXP3^+^ Treg [13,14,15]. Without BCL6 expression in the blood, FOXP3^+^ BCL6^−^ pTFR cells are more Treg-like and less TFH-like because BCL6 directs the differentiation of TFH cells. Additionally, even though pTFR cells express CXCR5 which distinguishes them from natural Treg, pTFR cells potentially could lose CXCR5 upon activation [31]. Therefore a functional assay for pTFR cells is needed in order to confirm their suppressive function. If pTFR cells have the suppressive properties of lymphoid-tissue TFR cells [8], pTFR cells could be the precursor of lymphoid-tissue GC TFR cells.

Most importantly, we hypothesized that TFR cells are a potential candidate for HIV reservoirs because TFH cells, which previously included TFR cells, contribute to HIV persistence [32]. Viral reservoirs are a challenge in achieving a functional cure for HIV infection. Even when an individual receives successful cART treatment and has undetected viremia, HIV can still persist in certain tissues and resurface in the blood because of latent infection. Our findings suggest that pTFR cells can harbor HIV and contribute to HIV persistence because of their constitutive expression of the HIV co-receptor CCR5. An increase of CCR5 on pTFR cells has been observed after HIV infection, which may be the result of HIV-induced inflammation with elevated type I interferons [33]. However, based on the fluidity of blood and the minimal cell interactions, there might not be enough contacts for the virus to infect the pTFR cells in blood. HIV infection occurs mostly in the lymph nodes due to a higher cell density and a closer cell-to-cell contact. Further characterization and testing for the presence of HIV-Gag protein, will allow for further interpretation of these data. The presence of pTFH and pTFR cells in the blood may be a proxy measure for the condition of the lymph nodes during HIV infection, allowing for potential therapeutic applications. Thus, understanding the immunophenotype and function of pTFR cells and their connection to lymphoid-tissue GC TFR cells can yield insight into HIV persistence in HIV-infected participants.

Lastly, we have yet to find a surface marker that can substitute for the intracellular marker FOXP3 in order to identify pTFR cells for cell-sorting. Nevertheless, our data showed that HIV infection affects the phenotypic characteristics of pTFH and pTFR cells. First, CCR5, an activation marker and HIV co-receptor, was upregulated in HIV infection, similar to high level of CCR5 expression in tonsillar TFR cells [34]. Secondly, TIGIT expression on pTFR cells was similar in both HIV-infected and non-infected participants, suggesting that these cells are not exhausted and can potentially suppress pTFH cells in an in vitro suppression assay. Thirdly, PD-1 expression on frozen cells was reduced significantly compared to fresh cells. Although freezing/thawing did affect PD-1 dramatically, where detected, the frequency of PD-1^+^ pTFH and pTFR cells showed a trend of increasing in HIV infection. In mice, PD-1 signals repressed TFR cells in peripheral blood [35]. This suggested that more PD-1 expression in HIV infection can affect the function of TFR cells. Fourthly, 5% of TFR cells expressed ICOS constitutively (as compared to CD3^+^CXCR5^+^ T cells). In influenza infection, ICOS stimulation is needed to maintain FOXP3 expression in the absence of IL-2 [36]. In mice, ICOS-expressing T cells produced IL-2, and a distinct population of memory phenotype CD4^+^ T cells constitutively expressed ICOS [37]. In human pTFR cells, ICOS might have a similar role in producing IL-2 in order to maintain FOXP3 expression. It is also possible that this small population of ICOS^+^ TFR cells constitutes memory cells from the lymph nodes. Additionally, the increasing trend of ICOS expression in HIV-infected participants suggests that pTFR and pTFH cells are activated due to HIV infection because ICOS is expressed on activated CD4^+^ and CD8^+^ cells [38].

Overall, we showed that the only intracellular marker that pTFR cells express is FOXP3 and that pTFR cells do not express BCL6 with our current protocol for intracellular staining, in contrast to data from our laboratory showing FOXP3^+^ BCL6^+^ TFR in the tonsil (data not shown). These pTFR cells are uniquely different from the CD25^+^ C XCR5^−^ Treg even when frozen. We also attempted to delineate the difference in pTFR populations in HIV-infected and non-infected MACS participants by using the Helios Mass Cytometer (CyTOF)-based system. However, the results were not as reliable as traditional flow cytometry (Supplementary Materials Figure S1) because mass cytometry is less sensitive with respect to FOXP3 detection [39]. Furthermore, peripheral blood TFR (CD4^+^CXCR5^+^CD25^high^CD25^low^) and TFH (CD3^+^CD4^+^CXCR5^+^CD127^neg to dim^CD127^dim to bright^) cells were recently found to harbor a comparable level of HIV-1 DNA in HIV-positive patients [40]. Due to high levels of CCR5 expression, pTFR cells could potentially be infected by HIV and contribute to HIV persistence. Understanding TFR cells characteristics and function upon HIV infection can be a valuable step in the path toward developing a functional cure for HIV.

## Figures and Tables

**Table 1:** Flow cytometry-Antibodies and their sources.

Marker	Fluorochrome	Company	Clone	Cat. No.
**FOXP3**	FITC	eBioscience	236A/E7	11-4777-42
**IgG1**	Alexa Fluor^®^ 488	Invitrogen	P3.6.2.8.1	53-4714-42
**BCL6**	PE	BD Bioscience	K112-91	561 522
**IgG1**	PE	BD Bioscience	X40	349 043
**CCR5 (CD195)**	PE-CF594	BD Bioscience	2D7/CCR5	562 456
**IgG2a**	PE-CF594	BD Bioscience	MOPC-173	563 489
**TIGIT**	APC	eBioscience	MBSA43	17-9500-42
**Mouse IgG1**	APC	BD Bioscience	-	340 442
**CD3**	eVolve^TM^ 605	Invitrogen	OKT3	83-0037-42
**CD4**	APC-Cy7	BD Bioscience	RPA-T4	557 871
**CD8a**	eVolve^TM^ 655	eBioscience	53-6.7	86-0081-42
**CD25**	eFluor^®^ 450	eBioscience	BC96	48-0259-42
**CD45RA**	PerCP Cy5.5	eBioscience	HI100	45-0458-42
**CXCR5 (CD185)**	PE-Cy7	eBioscience	MU5UBEE	25-9185-42
**ICOS (CD278)**	APC	eBioscience	MBSA43	17-9948-42
**PD1 (CD279)**	APC	eBioscience	eBioJ105	17-2799-42
**Viability dye**	Violet ^TM^510	Tonbo	n/a	13-0870-T100

**Table 2:** CYTOF metal-isotype conjugated antibodies.

Label	Target	Clone
115In	Ki-67	SolA15
141Pr	TIGIT	MBSA43
142Nd	CD31/PECAM-1	WM59
143Nd	CD25 (IL-2R)	2A3
144Nd	CD69	FN50
146Nd	CD8a	RPA-T8
150Nd	CD223/LAG-3	11C3C65
153Eu	CD185 (CXCR5)	RF8B2
155Gd	CD279 (PD-1)	EH12.2H7
156Gd	CD184 (CXCR4)	12G5
159Tb	CD197 (CCR7)	G043H7
161Dy	CD152 (CTLA-4)	14D3
162Dy	FoxP3	PCH101
163Dy	BCL-6	K112-91
165Ho	CD19	HIB19
168Er	CD278/ICOS	C398.4A
169Tm	CD45RA	HI100
170Er	CD3	UCHT1
171Yb	CD195 (CCR5)	NP-6G4
172Yb	KC-57	
174Yb	CD4	SK3
176Yb	CD127 (IL-7Ra)	A019D5

## Data Availability

Data in this manuscript were collected by the Multicenter AIDS Cohort Study (MACS), now the MACS/WIHS Combined Cohort Study (MWCCS). The contents of this publication are solely the responsibility of the authors and do not represent the official views of the National Institutes of Health (NIH). Los Angeles MACS Center (Roger Detels and Matthew Mimiaga), U01-AI035040; Los Angeles MWCCS CRS (Roger Detels and Matthew Mimiaga), U01-HL146333; MWCCS Data Analysis and Coordination Center (Gypsyamber D’Souza, Stephen Gange, and Elizabeth Golub), U01-HL146193. The MWCCS is funded primarily by the National Heart, Lung, and Blood Institute (NHLBI), with additional co-funding from the Eunice Kennedy Shriver National Institute of Child Health and Human Development (NICHD), the National Institute on Aging (NIA), the National Institute of Dental and Craniofacial Research (NIDCR), the National Institute of Allergy and Infectious Diseases (NIAID), the National Institute of Neurological Disorders and Stroke (NINDS), the National Institute of Mental Health (NIMH), the National Institute on Drug Abuse (NIDA), the National Institute of Nursing Research (NINR), the National Cancer Institute (NCI), the National Institute on Alcohol Abuse and Alcoholism (NIAAA), the National Institute on Deafness and Other Communication Disorders (NIDCD), the National Institute of Diabetes and Digestive and Kidney Diseases (NIDDK), the National Institute on Minority Health and Health Disparities (NIMHD), and in coordination and alignment with the research priorities of the National Institutes of Health, the Office of AIDS Research (OAR). MWCCS data collection is also supported by UL1-TR001881 (UCLA CTSI). Flow Cytometry data can be provided upon request.

## Supplementary Materials

The following supporting information can be downloaded at: https://www.mdpi.com/article/10.3390/cells12020296/s1, Figure S1: General gating strategy for the detection of TFH and TFR in PBMC. Figure S2: TFR are present in PBMC from non-infected and HIV-infected individuals using Mass Cytometry.
