# Supplementary material for: Characterization of T Follicular Helper Cells and T Follicular Regulatory Cells in HIV-Infected and Sero-Negative Individuals
Source: Cells. 2023 Jan 12;12(2):296. doi: 10.3390/cells12020296 (PMC9856637; doi:10.3390/cells12020296)
Supplement: Supplementary file 1 [file cells-12-00296-s001.zip › cells-1845384-supplementary.pdf]

Supplementary Materials:

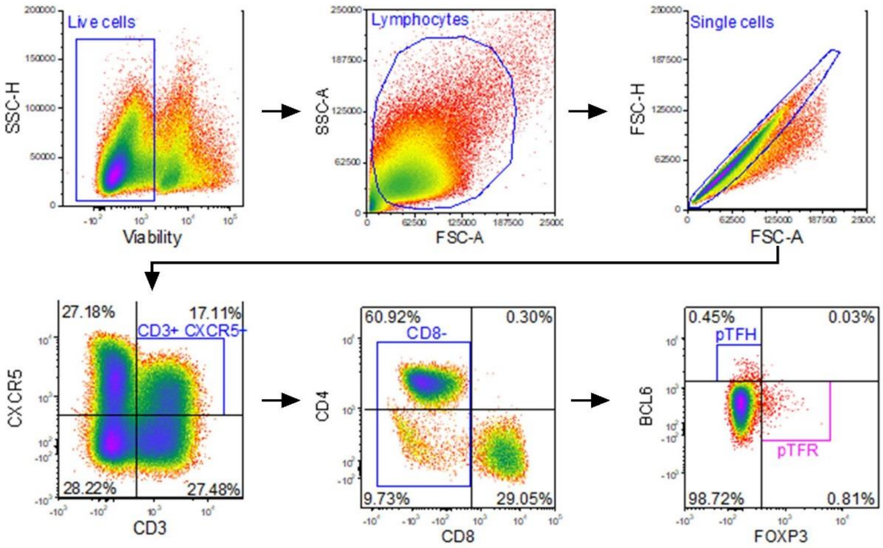

Figure S1. General gating strategy for the detection of TFH and TFR in PBMC.

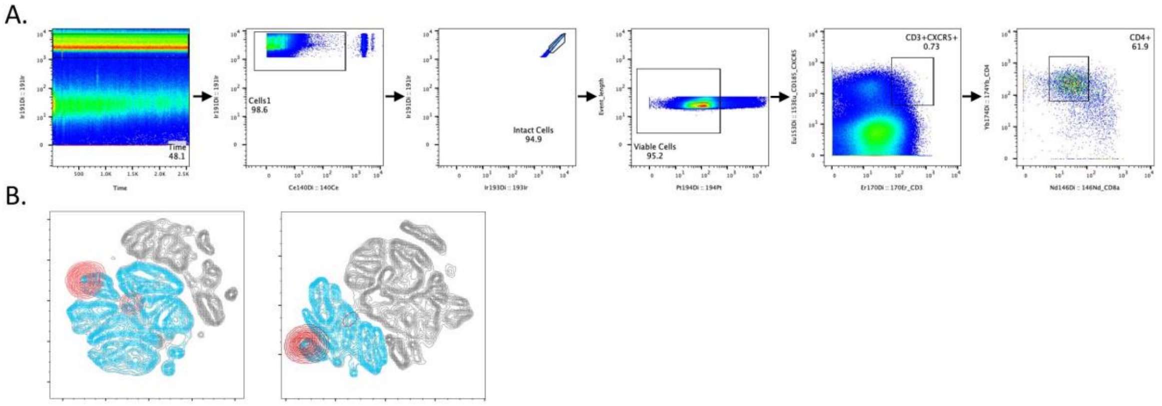

Figure S2. TFR are present in PBMC from non-infected and HIV-infected individuals using Mass Cytometry. (**A**) Gating strategy. (**B**) PBMC from non-infected (left) and HIV infected (right) individuals were immunophenotyped using Mass Cytometry (CyTOF). FiTSNE plots of T cells (CD3+CD19-) show that CD3+CXCR5+CD19-FOXP3+ cells TFR (Red) are present in the CD4+ population (Blue), but not in the CD8+ population (Grey).
